# BIOMARKERS OF AGING

**DOI:** 10.1093/geroni/igae098.0982

**Published:** 2024-12-31

**Authors:** Chhanda Dutta

**Affiliations:** Chair

## Abstract

It may be possible to prevent or ameliorate multiple adverse age-related outcomes through interventions targeted at fundamental mechanisms of aging, such as cell senescence, proteostasis, autophagy, mitochondrial dysfunction, macromolecular damage, and epigenetic regulation. Understanding the relationships between aging mechanisms and aging conditions as a basis for identifying targets for interventions to extend healthy life span has been a long-standing goal of biomedical aging research. The identification of sufficiently valid, reliable, and accurate aging mechanistic markers in humans would greatly facilitate the ability of observational studies to clarify the relationships of aging markers to aging-related phenotypes and enhance geroscience-based therapeutics development. To take full advantage of such aging mechanistic markers in future translational aging research, it will be important to clarify the degree of correlation between a) circulating levels and their activity in tissues/organs, b) their activity in laboratory animals and in humans, and c) whether they can capture the status of a specific mechanism across the life span, as part of the development and validation process. This session will feature presentations which highlight advances in the development of aging mechanistic markers, as well as challenges and limitations. Dr. Birgit Schilling will discuss the utility of specific markers of cellular senescence and multi-omics biomarkers. Dr. Nathan LeBrasseur will talk about the use of aging mechanistic markers for predicting clinical outcomes and selection of patient populations for clinical interventions. Dr. Anthony Molina will present on the application of blood-based bioenergetic profiling to elucidate the role of mitochondrial dysfunction in different aging conditions.

